# New Phenotype and Mineralization of Biogenic Iron Oxide in Magnetotactic Bacteria

**DOI:** 10.3390/nano11123189

**Published:** 2021-11-25

**Authors:** Walid Baaziz, Corneliu Ghica, Jefferson Cypriano, Fernanda Abreu, Karine Anselme, Ovidiu Ersen, Marcos Farina, Jacques Werckmann

**Affiliations:** 1 Institut de Physique et Chimie des Matériaux de Strasbourg (IPCMS), University of Strasbourg, 23 rue du Loess BP 43, CEDEX 2, 67034 Strasbourg, France; walid.baaziz@ipcms.unistra.fr (W.B.); ovidiu.ersen@ipcms.unistra.fr (O.E.); 2 National Institute of Materials Physics, Atomistilor 405A, 077125 Magurele, Romania; 3 Instituto de Microbiologia Paulo de Góes, Universidade Federal do Rio de Janeiro, Avenida Carlos Chagas Filho, 373, CCS, UFRJ, Rio de Janeiro 21941-902, Brazil; jeffcy@micro.ufrj.br (J.C.); fernandaaabreu@micro.ufrj.br (F.A.); 4 Institut de Science des Matériaux de Mulhouse, University of Haute Alsace, 68057 Mulhouse, France; Karine.Anselme@uha.fr; 5 Instituto de Ciências Biomédicas, Universidade Federal do Rio de Janeiro, Rio de Janeiro 21941-902, Brazil; marcos.farina.souza@gmail.com; 6 Centro Brasiliero de Pesquisas Fisicas, LABNANO, Rio de Janeiro 22290-180, Brazil

**Keywords:** magnetotactic bacteria, phenotype, magnetite, maghemite, electron microscopy, EDS, electron tomography, high-resolution imaging

## Abstract

Many magnetotactic bacteria (MTB) biomineralize magnetite crystals that nucleate and grow inside intracellular membranous vesicles originating from invaginations of the cytoplasmic membrane. The crystals together with their surrounding membranes are referred to as magnetosomes. Magnetosome magnetite crystals nucleate and grow using iron transported inside the vesicle by specific proteins. Here, we tackle the question of the organization of magnetosomes, which are always described as constituted by linear chains of nanocrystals. In addition, it is commonly accepted that the iron oxide nanocrystals are in the magnetite-based phase. We show, in the case of a wild species of coccus-type bacterium, that there is a double organization of the magnetosomes, relatively perpendicular to each other, and that the nanocrystals are in fact maghemite. These findings were obtained, respectively, by using electron tomography of whole mounts of cells directly from the environment and high-resolution transmission electron microscopy and diffraction. Structure simulations were performed with the MacTempas software. This study opens new perspectives on the diversity of phenotypes within MTBs and allows to envisage other mechanisms of nucleation and formation of biogenic iron oxide crystals.

## 1. Introduction

Since their observation and description by transmission electron microscopy by Blakemore in 1975 [1], magnetotactic bacteria (MTB) have given rise to numerous works and publications until today. The great interest for these organisms is due to their structure and properties induced by biomineralized magnetic nanoparticles present inside the cell body. It is indeed a sufficiently simple organism which allows genetic manipulations opening the way on one hand to the understanding of the mechanism of biomineralization [2] and on the other hand to their potential applications in the field of biomedicine [3,4] and paleomagnetism [5]. MTB are ubiquitous microorganisms observed in all types of aquatic environments [6].

MTB synthesizes chains of nano-sized, membrane-bound, iron-rich magnetic mineral crystals [7]. Each crystal with its associated membrane is called a magnetosome (MS). These intracellular chains of organelles are either composed of magnetite (Fe_3_O_4_) or greigite (Fe_3_S_4_) [8,9]. Furthermore, Wali et al. hypothezized that bacteria producing magnetite could transform their magnetosomes’ mineral into the ferrimagnetic spinel, maghemite [10]. These intracellular chains of organelles impart to the organism a sufficiently large magnetic moment to allow for the passive alignment of the bacteria in the Earth’s geomagnetic field. This passive alignment associated with active swimming modulated by aerotaxis is responsible for the localization and positioning of MTB at the optimal region of the oxic–anoxic transition zone, in the sediment and water where they thrive [11]. In MTB, the magnetosome biomineralization process is under strict biochemical control [2,12]. Specific genes/proteins are involved in the biomineralization of the magnetosomes in chains [13,14]. Most genes involved in magnetosome formation are called *mam* (magnetosome membrane) or *mms* (magnetic particle membrane-specific) genes, and are usually clustered in a relatively large, single chromosomal region in the genome. Numerous studies have shown that the formation of MS is governed by the joint action of several proteins [13,15]. A mechanism divided into three stages has been proposed for the MS formation: bacteria membrane invagination, iron uptake, and magnetite particle biomineralization [14]. Studies to understand the magnetosomes’ formation mechanism have been performed on MTB which are easily cultivated, such as the *Magnetospirillum* strain AMB-1 [16,17] and the *Magnetospirillum gryphiswaldense* strain MSR-1 [18], which show the particularity of having a magnetosome chain organized by a filamentous network that runs parallel to the cell membrane and joins the two poles of the cell. Our study focuses on the analysis of whole mounts of a wild, coccus-type bacterium, whose internal architecture of magnetosomes is unknown. They synthesize magnetosomes that appear as clusters rather than chains after their deposition onto thin films and whose iron oxide nanocrystals have a nearly perfect prismatic morphology. Thanks to electron tomography, we show that these clusters have a specific organization. By analyzing structural information obtained from high-resolution transmission electron microscopy (HRTEM), we show that iron oxide nanocrystals are composed of maghemite, which is an iron-deficient magnetite. This type of bacteria also has the particularity of containing large granules of polyphosphate, whose composition depends on the elements dissolved in the water column in which they live [19].

## 2. Materials and Methods

### 2.1. Sampling and Collection of MTB

The sediments were collected on the lagoon beach. They are 30 cm from the surface and are deposited to a thickness of about 10 cm on a stony bottom. A first filtration was carried out to allow only sediments with a size of less than 2 mm to pass. They were stored in a 1 L bottle with a 1/L volume proportion. The respective masses of sediment and water have not been evaluated. They were kept for 1 month in natural light. The collected magnetotactic bacteria were magnetically enriched by attaching the south pole of a permanent magnet to the outside of the bottle. They were collected in a Becher containing filtered water (0.2 µm) and purified by the same method. The whole cells were directly deposited on 300-mesh gold-coper lacey carbon grids (Ted Pella, Inc., Redding, CA, USA) and observed after drying. The grids were kept in a dehydrated box. Electron tomography was performed a month later. Eighteen months later, high-resolution transmission electron microscopy (HTREM) and energy dispersive spectroscopy (EDS) analysis observations were performed.

### 2.2. Electron Microscopy

For the X-ray chemical mapping, an ASTEM JEOL JEM-ARM200F Cold FEG (JEOL, Tokyo, Japan) operating at 200 kV in scanning transmission electron microscopy (STEM) mode, equipped with a high-sensitivity energy dispersive X-ray spectroscopy (EDXS) setup characterized by 1 steradian large solid-angle silicon drift detector (SDD), was used. For electron tomography (ET), the acquisition of tilt series was performed using a JEOL2100F microscope (JEOL, Tokyo, Japan) equipped with a CS corrector operating at 200 kV in STEM mode, via the tomography plugin of the Digital Micrograph software. The ADF (annular dark field) and BF (bright field) tilt series in the STEM were recorded by using the ADF and BF detectors. The specimen was tilted in the angular range of +74° and −70° with an increment of 2° in the equal mode. The inner radius of the ADF detector was about 40 mrad, a relatively large value that allows us to consider that the intensity in the corresponding images is in a first approximation proportional to the mean atomic number of the specimen. The recorded images were spatially aligned by cross-correlating consecutive images using IMOD software (University of Colorado, Boulder, CO, USA). The volume calculation was realized using the algebraic reconstruction technique (ART) implemented in the TomoJ plugin working in the ImageJ software (National Institute of Health, Bethesda, MD, USA). Finally, the visualization and the analysis of the obtained volumes were carried out using the displaying capabilities and the iso-surface rendering method in the 3DSlicer software (Brigham and Women’s Hospital, Boston, MA, USA). The atomic structure of the magnetosomes was also analyzed by HRTEM using a JEM ARM 200F (JEOL, Tokyo, Japan) instrument operating at 200 kV (point-to-point resolution 0.18 nm) and the MacTempas software (version 2.4.25, Berkeley, CA, USA) for image and electron diffraction pattern simulations. 3D crystal models were constructed using KrystalShaper software (JCrystalSoft, http://www.jcrystal.com/).

## 3. Results and Discussion

### 3.1. Phenotype and Crystal Morphology

**Figure 1:**
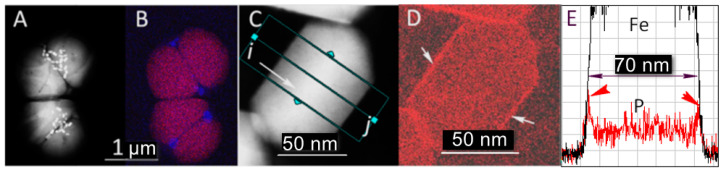
Morphology and chemical map of some representative parts of the analyzed specimen. (**A**) Dark-field image of two adjacent bacteria. (**B**) Superposition of chemical maps of P (red) and S (blue). (**C**) High-resolution image of a crystal with near-perfect morphology. The green rectangle is the area of accumulation of the Kα signal of the P from i to j, in order to obtain the graph (**E**). (**D**) Map of P. The white arrows indicate the presence of P on the contour of the crystal. (**E**) Spatial variation of the intensity collected from i to j. In red, the intensity of P, which has a peak within the region delimited by red arrows, corresponding to the localization of iron (in black).

**Figure 2:**
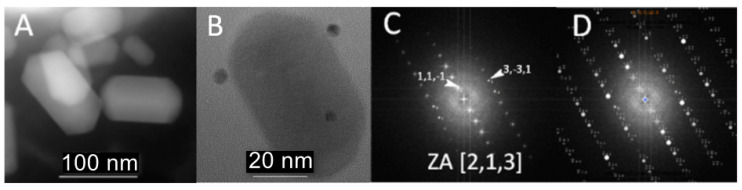
Structure and analysis of the specimen. (**A**) Random arrangement of crystals with an acicular prismatic morphology. (**B**) High-resolution imaging: the dark dots are gold nanoparticles used as fiducial markers for tomography. (**C**) FFT micrograph of the image in (**B**): the zone axis of the crystal is parallel to the <111> crystallographic direction. (**D**) Simulated diffraction pattern.

The wild-type bacteria collected were studied by analytical scanning transmission electron microscopy (ASTEM) using a focused electron beam to determine their general morphology and to map key elements that constitute the bacteria: Fe, O, P, and S. Two adjacent bacteria are shown (Figure 1A,B), that consist of two large globules of polyphosphate, composed in addition to P and O, Na, Mg, and K (Supplementary Figure S1), which occupy almost the entire volume of the bacteria. The iron oxide nanocrystals appear to be randomly distributed near the interface between the two phosphate globules (Figure 1A). The crystals are large and can reach up to 120 nm in length (Figure 2A). They are of pseudohexagonal prismatic shape, which is one of the characteristic shapes of biogenic iron oxides in the cubic system [20,21] The regular shape and size of magnetosomes’ crystals indicate that they were mature. Phosphate-rich ferric hydroxide phase corresponding to the early stage of magnetosome formation in *M. magneticum* strain AMB-1 [22] were not observed in the analyzed samples. The mapping of the P carried out in high-resolution imaging mode using an electron beam with lateral size of less than 0.5 nm makes it possible to demonstrate the magnetosomes’ phospholipid envelope (Figure 1D,E). This envelope corresponds to the vesicle in which the iron oxide nanocrystals nucleate and grow. The phase images obtained when the microscope operates in high resolution in parallel mode (Figure 2B) allow us to assign to the crystals either the structure of magnetite or that of maghemite. In both cases, the direction <111> corresponds to the major axis of the crystal (Figure 2C,D).

**Figure 3:**
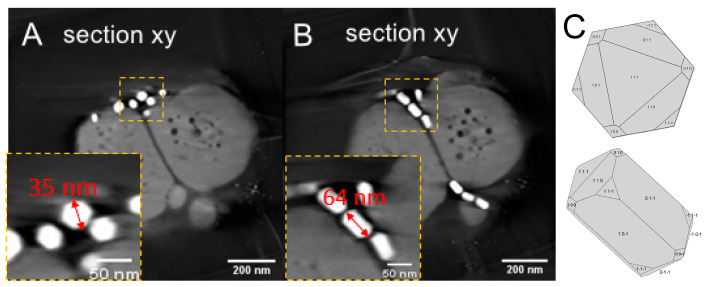
Spatial distribution of the magnetosomes and morphology of the crystals. (**A**) Section extracted from the reconstruction at a given depth. The perpendicular section of the crystal depicting a hexagonal contour is highlighted in the insert. (**B**) Section taken at another depth; insert, parallel section of a crystal. (**C**) Idealized model based on the observations from tomography and HRTEM images.

The spatial distribution and the morphology of the putative magnetite crystals were obtained from the tomography. Figure 3A, taken at 0° tilt, presents a projected overview of the bacteria. Two magnetosome groups are highlighted by extracting tomographic slices at two different depths, Z1 and Z2 (Figure 3A,B). One group contains magnetosomes oriented with their long axis perpendicular to the plane of the figure (Figure 3A), which is that of the support grid, while in the other (Figure 3B), they are parallel to the observation plane. These two slices provide a complete view of the morphology of the crystals. In fact, the insert of Figure 3A shows a perpendicular section of a crystal, perfectly hexagonal with respect to the planar morphology of the horizontal section in the insert of Figure 3B. This confirms the pseudohexagonal prismatic morphology of these crystals.

**Figure 4:**
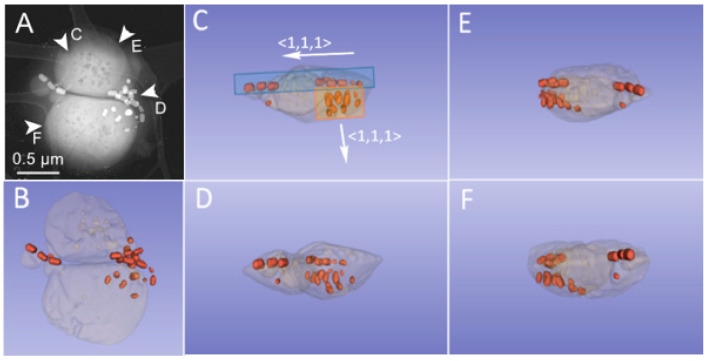
3D representation of the crystals in the bacteria. (**A**) Dark-field image at 0° tilt angle. (**B**–**F**) Images corresponding to the viewing directions of the 3D representation indicated by white arrows in (**A**). The white arrows in (**C**) indicate the crystallographic orientation of the crystals.

The 3D distribution of the magnetosome and the crystals’ *c* axes <111> are presented in Figure 4 through a 3D representation of the calculated reconstructions. Figure 4C–F present different orientations according the direction of the tomographic volume indicated by the white arrows in Figure 4A. Two orientations perpendicular to each other of the magnetosomes and crystals are highlighted. The blue and red rectangles in Figure 4C illustrate this organization. The crystallographic directions <111> of the crystals are therefore also perpendicular to each other, as indicated by the arrows in Figure 4C.

MTB are recognized as the simplest organisms that display geomagnetic field orientation, apparently using it to increase the efficiency of chemotaxis in locating and maintaining an optimal position where both electron donors and acceptors are available to the cells [23]. This new spatial configuration of the magnetosomes contained in this bacterium opens the possibility of an evolutionary advantage. Although we cannot discard the possibility that the organization pattern of magnetosomes inside the analyzed bacteria is a consequence of internal forces due to the air-drying process, it will be interesting to characterize the movement of this bacterium in the environment [24] and to analyze the 3D distribution of magnetosomes by cryotomography [25].

### 3.2. Crystallographic Characterization

**Figure 5:**
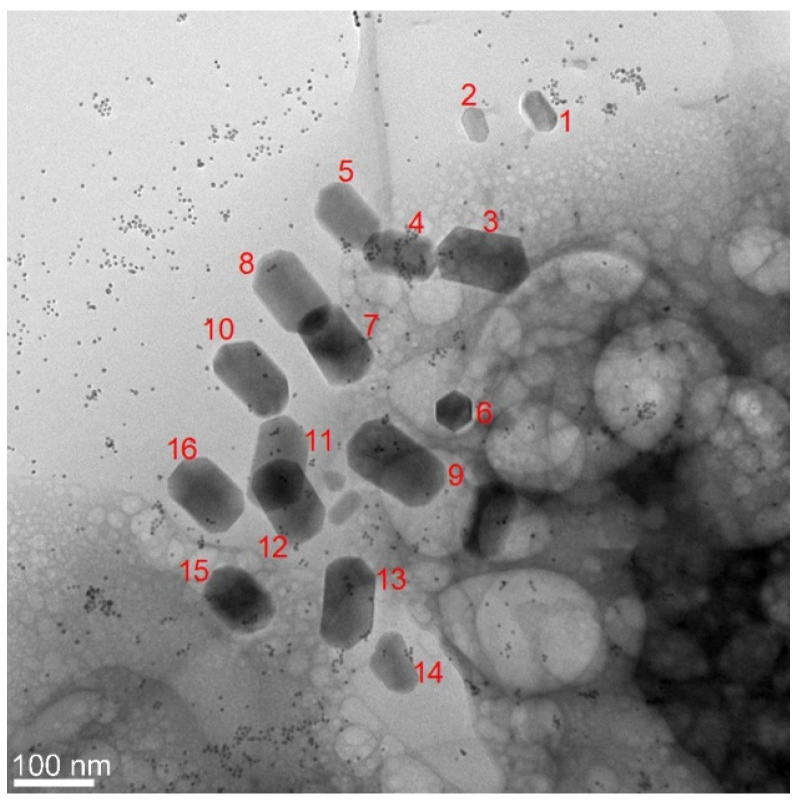
Low-magnification TEM image of a typical assembly of magnetosomes mineralized inside a magnetotactic bacterium.

While it is widely accepted that the crystal structure of the iron-based magnetosomes is that of magnetite (Fe_3_O_4_, spinel structure), our study was also intended to check whether other types of iron oxides may result through the biomineralization process, with maghemite (γFe_2_O_3_) being one of the main candidates [4]. In the low-magnification TEM images acquired from our samples, the iron-based magnetosomes can be typically found as ensembles of faceted elongated grains with a size of up to 120 nm, often lined up in short chains along their long axis (Figure 5). Several HRTEM micrographs have been recorded from different magnetosomes observed inside the same or different bacteria. For the identification of their structure, we have analyzed the FFT patterns of the recorded HRTEM micrographs and the atomic-resolution patterns of the zone-axis-oriented magnetosomes.

**Figure 6:**
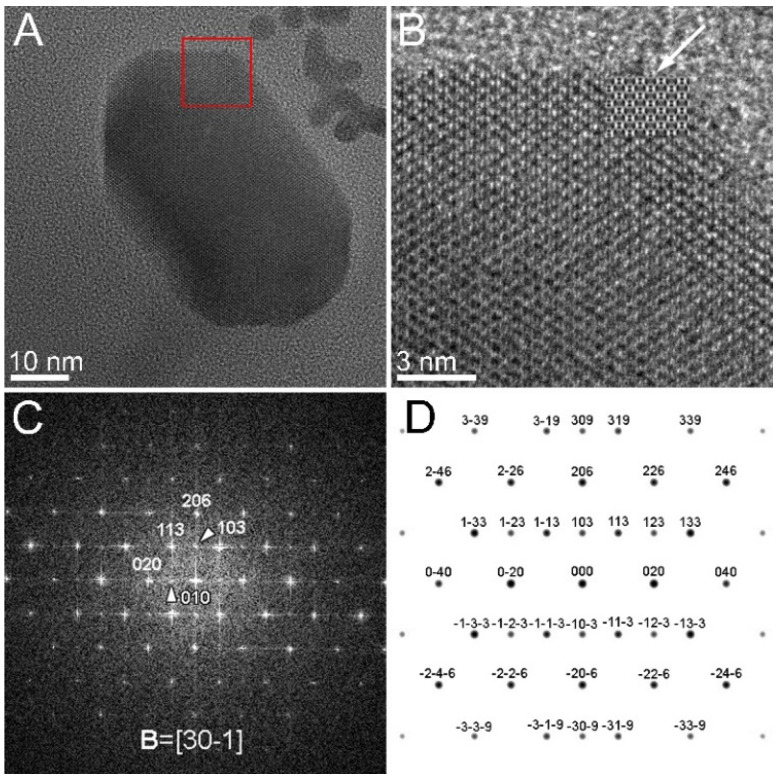
(**A**) HRTEM micrograph of the nanograin denoted by “1” in Figure 5. (**B**) Enlarged image of the upper-right corner of the nanograin showing the detailed HRTEM pattern. (**C**) FFT micrograph corresponding to the area delimited by the red square in (**A**). (**D**) Simulated SAED pattern considering the tetragonal unit cell of maghemite (cif No. 9006318).

The HRTEM micrograph in Figure 6A represents the nanograin denoted by “1” in Figure 5, taken at higher magnification. The enlarged HRTEM pattern (Figure 6B) and the associated FFT pattern (Figure 6C) correspond to the area delimited by the white square in Figure 6A. The attempt of indexing the FFT pattern according to the magnetite crystal structure failed due to the presence of intensity spots in forbidden positions, marked with white arrowheads, which suggests possible structural/stoichiometry defects. The presence of vacancies could be a cause for the imbalance of the magnetite structure factor, pointing to the related structure of maghemite. Compositional variations during the biomineralization of magnetite magnetosomes have been mentioned in several other studies as being induced by the growth conditions, such as environmental oxygen concentrations. Increased oxygen supply may result in the production of more non-stoichiometric magnetite in magnetosomes [17]. Moreover, in a study devoted to the incorporation of Co^2+^ ions in magnetosomes by replacing the Fe^2+^ ions in octahedral sites of magnetite, it was noticed that the composition of the magnetosomes produced by the AMB-1 cells in the Co-free culture slightly deviated from stoichiometric magnetite, most likely due to crystal defects (e.g., cation vacancy) occurring during magnetosome biomineralization. The experimental X-ray magnetic circular dichroism (XMCD) spectra at Fe L_2,3_ edges were fitted by a linear combination of the experimental XMCD signal for pure magnetite and pure maghemite [25]. In our case, the presence of defects such as vacancies may have an impact on the HRTEM pattern of the magnetosomes in certain orientations. It means that in such particular orientations, such as the one in Figure 6C, the indexation of the FFT pattern becomes indeed feasible only by considering the tetragonal unit cell of γFe_2_O_3_ (maghemite, cif No. 9006318), which can be described as a defected spinel structure [26] with cationic vacancies located in 1/6 of the octahedral positions.

**Figure 7:**
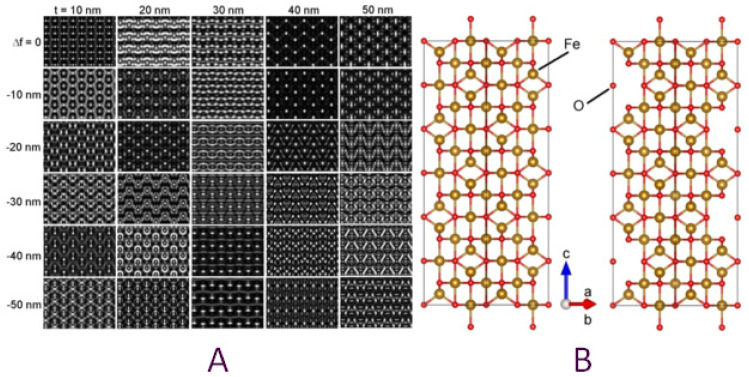
(**A**) Matrix of simulated HRTEM patterns based on the tetragonal unit cell of maghemite in B = [30–1] orientation. (**B**) Structural model of the tetragonal unit cell of maghemite (right-hand side) viewed along the [1–10] crystallographic direction revealing the vacant octahedral Fe positions with respect to the same structure with fully occupied atomic positions (magnetite).

The way in which vacancies are distributed among the octahedral positions generates three variants of maghemite with three different symmetries. In the case of a totally random distribution of the Fe vacancies over the octahedral positions, a maghemite (Fe_2_O_3_) structure having an Fd-3m symmetry (S.G. No. 227) was obtained, just as for a perfect (free of vacancies) spinel structure such as magnetite (Fe_3_O_4_). If the Fe vacancies are ordered in some particular octahedral positions, two different symmetries can be generated, namely a cubic primitive (S.G. No. 213, P4_1_32) lattice, keeping the same lattice parameter (*a* = 0.833 nm), or a tetragonal one (S.G. No. 96, P4_3_2_1_2) [26]. The tetragonal unit cell has a *c* lattice parameter three times that of the spinel one (*a* = *b* = 0.8330 nm, *c* = 2.4990 nm), and ordered Fe vacancies placed in the 5/8, 3/8, 2/24 octahedral positions (8b Wyckoff position). Some of the analyzed diffraction patterns could only be explained by using the tetragonal unit cell, which we further considered for additional confirmation through image and diffraction pattern simulation software. The graphical representation of the tetragonal unit cell is depicted in Figure 7B, where the vacancy-containing cell (right-hand side) is shown along the [1–10] direction next to a fully populated one (3 magnetite cells piled up), so that the Fe vacancies can be clearly noticed. We used the vacancy-containing tetragonal unit cell and the Mac Tempas simulation program to generate HRTEM and SAED patterns of the tetragonal maghemite in the [30–1] orientation, as indexed in Figure 6C. The series of simulated HRTEM patterns are displayed in Figure 7A for 5 different thicknesses and 6 under-focus values, ranging from 10 to 50 nm and 0 to −50 nm, respectively. 

Comparing the simulated HRTEM patterns with the experimental micrograph in Figure 6B, one can notice that the pattern corresponding to a thickness of 10 nm and a defocus of −20 nm fits very well with the experimental image in the thinnest regions of the grain. For a better comparison, the mentioned simulated pattern has been inserted in the upper-right corner of the grain, as pointed out by the white arrow.

Moreover, the simulated SAED pattern represented in Figure 6D perfectly fits the FFT distribution in Figure 6C, as the 103 spot is now allowed by the symmetry conditions ruling the vacancy-containing tetragonal structure. The presence of the 103 spot also allows for explaining the faint 010 spot by a double diffraction event on the (113) and (−10–3) planes. We used the same tetragonal structure for the successful interpretation of the HRTEM images and the associated FFT patterns observed in the case of other grains in Figure 5, such as the ones numbered as 2, 4, or 5. Additional examples of nanograins for which the maghemite structure is more suitable for explaining the HRTEM/FFT patterns are provided in the Supplementary Materials (Figures S2–S4). Given the structure similarity between magnetite and maghemite, only a limited number of crystal orientations enable the observation of those details (usually additional faint spots), helping to differentiate the two structures. In some other cases, the grain orientation (far from a zone axis) did not allow us to come to a clear conclusion as to their crystalline structure, since the observed lattice fringes and the associated FFT patterns could be indexed either as magnetite or maghemite.

## 4. Conclusions

To understand the process of magnetosome formation and distribution inside cells, we have carried out a 3D study by electron tomography on cultivable species with elongated magnetosomes and magnetosome chains parallel to the long axis of bacteria [16,25]. We showed the importance of such a study on non-cultivated bacteria obtained directly from the environment [27], as some aspects that have been generalized from cultivated species deserve revision. We highlighted two examples: the identification of the mineral content of magnetosomes and the 3D organization pattern of magnetosomes within the MTBs. Since the first observations made by Blakemore, it was commonly accepted that the biogenic iron oxide synthesized by MTBs is magnetite. Our results show that this idea will certainly have to be revised by developing local studies by high-resolution electron microscopy, because it is very probable that maghemite can also be present, paving the way for new interpretations on the nucleation and growth mechanisms of iron oxides within MTBs. From this perspective, maghemite formation could be a result of accessory biological functions of magnetosomes that have been reported for magnetosomes, which are in the scope of the protective effect against reactive oxygen species or metal stress [28,29,30]. The other example concerns the determination of the way magnetosomes arrange inside bacteria, as it imparts a total magnetic moment to the organism, with direct consequences in their trajectories under applied magnetic fields. This subject would greatly benefit from electron tomography, as well as the collection of bacteria from the environment. Besides, this procedure would also contribute to evaluate the possible relationship between the morphology of individual crystals and the way in which they are organized in the chains. The two main points focused on in this work can significantly contribute to various areas of research, such as biosignatures (identification of biogenic magnetic nanocrystals in soils, meteorites, or materials from planetary origin) and the biological response to applied magnetic fields, helping in the construction of magnetic sensors based on different spatial configurations of their magnetic single-domain nanocomponents.

## Data Availability

The data presented in this study are available on request from the corresponding authors Jacques Werckmann.

## Supplementary Materials

The following are available online at https://www.mdpi.com/article/10.3390/nano11123189/s1, Figure S1: EDX analysis. (A) Analysis of the region circled in red. (B) EDSX spectrum obtained. (C) Scanning of i a j to collect the evolution of signals across the interface of contiguous bacteria; (D,E) evolution of the intensity of the signals coming from the elements present; Figure S2: (a) HRTEM micrograph of a magnetosome and (b) the enlarged image corresponding to the dashed-line square in (a) showing the HR lattice planes; (c) FFT diagram from a large area on the grain in (a) containing faint spots (pointed by tilted arrows) in positions that are not allowed for the magnetite structure; (d) enlargement of the FFT in (c) showing the faint spots in forbidden positions; (e) Orientation of the line profile through the FFT maxima in (c) and (f) intensity of the FFT maxima along the line profile; Figure S3: (a) HRTEM micrograph of a magnetosome and (b) the enlarged image from the left-hand border of the grain (a) showing the HR lattice planes; (c) FFT diagram from a large area on the grain; the calculated interplanar distances in nm are indicated on the left side of the diagram and the Miller indices according to the maghemite structure on the right side of the diagram; the crystal orientation corresponds to B = [−211]; (d) the atomic structural model of the tetragonal maghemite in [−211] orientation (obtained with VESTA); Figure S4: (a) HRTEM micrograph of a magnetosome and (b) the associated FFT diagram indexed according to the maghemite structure (B = [−110]); (c) intensity profile along a line crossing the hh0 spots; (d) the atomic structural model of maghemite in [−110] orientation (using VESTA); (e) simulated electron diffraction pattern using the maghemite structure in [−110] orientation; Figure S4: EDX analysis. (A) Analysis of the region circled in red. (B) EDSX spectrum obtained. (C) Scanning of i a j to collect the evolution of signals across the interface of contiguous bacteria; (D,E) evolution of the intensity of the signals coming from the elements present.
