# Supplementary material for: New Phenotype and Mineralization of Biogenic Iron Oxide in Magnetotactic Bacteria
Source: Nanomaterials (Basel). 2021 Nov 25;11(12):3189. doi: 10.3390/nano11123189 (PMC8706698; doi:10.3390/nano11123189)
Supplement: Supplementary file 1 [file nanomaterials-11-03189-s001.zip › nanomaterials-1450629-Supplementary Materials (new).pdf]

nanomaterials

MDPI

Supplementary information

New Phenotype and Mineralization of Biogenic Iron Oxide in Magnetotactic Bacteria

Walid Baaziz [1], Corneliu Ghica [2,*], Jefferson Cypriano [3], Fernanda Abreu [3], Karine Anselme [4], Ovidiu Ersen [1], Marcos Farina [5] and Jacques Werckmann [5,6,*]

1. Institut de Physique et Chimie des Matériaux de Strasbourg (IPCMS), University of Strasbourg, 23 rue du Loess BP 43, 67034 Strasbourg CEDEX 2, France; walid.baaziz@ipcms.unistra.fr (W.B.); ovidiu.ersen@ipcms.unistra.fr (O.E.)
2. National Institute of Materials Physics, Atomistilor 405A, 077125 Magurele, Romania
3. Instituto de Microbiologia Paulo de Góes, Universidade Federal do Rio de Janeiro, Avenida Carlos Chagas Filho, 373, CCS, UFRJ, Rio de Janeiro, RJ 21941-902, Brazil; jeffcy@micro.ufrj.br (J.C.); fernandaaabreu@micro.ufrj.br (F.A.)
4. Institut de Science des Matériaux de Mulhouse, University of Haute Alsace, 68057 Mulhouse, France; Karine.Anselme@uha.fr
5. Instituto de Ciências Biomédicas, Universidade Federal do Rio de Janeiro, Rio de Janeiro 21941-902, Brazil; marcos.farina.souza@gmail.com
6. Centro Brasiliero de Pesquisas Fisicas, LABNANO, Rio de Janeiro 22290-180, Brazil

* Correspondence: cghica@infim.ro (C.G.); j.werckmann@gmail.com (J.W.)

Elemental mapping

It has been shown that the magnetococcaceae bacteria play an important role in the phosphate cycle and that the large granules they contain are polyphosphates, and the composition of these granules is dependent on the chemical composition of the aquatic environment (S. Rivas-Lamelo et al. Magnetotactic bacteria as a new model for P sequestration in the ferruginous Lake Pavin, Geochem. Persp. Let. 5, 35–41 (2017).

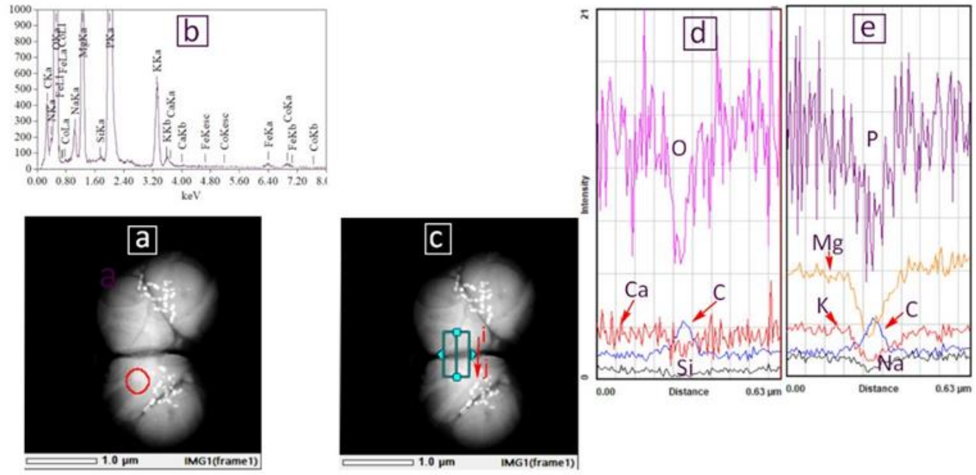

Figure S1. EDX analysis. (a) Analysis of the region circled in red. (b) EDSX spectrum obtained. (c) Scanning of i a j to collect the evolution of signals across the interface of contiguous bacteria; (d, e) evolution of the intensity of the signals coming from the elements present.

In Figure S1a the red circle indicates the domain from which the EDXS signals are extracted. Figure S1b, the peaks observed are those of C, N, Na, Mg, P, K, as well as the peaks Fe and Co. These last two signals come mainly from the interactions of X-ray and backscattered electrons on the lenses of the microscope (William and Carter e-ISBN 978-

0-387-76501-3) in the absence of iron the two peaks have the same height. The peak of iron is slightly larger than that of Co. But given the noise of the measurement, it cannot be concluded that there are traces of iron in the granule. In addition to be sure that the signals come from the granules and not from the outer membrane of the bacteria, a scan was carried out which crosses the contact interface between the two bacteria (Figure S1c).

It is observed that only the carbon peak increases at the interface because the beam passes through a great thickness of the outer membrane. No other element follows the shape of C's signal; they decrease sharply at the interface. The signals of Ca and Si correspond to noise.

The polyphosphate is composed mainly of O, P, Mg, Na and K (Figure S1d,e)

Crystal structure analysis by HRTEM

Although the two minerals, magnetite and maghemite, have similar crystal structures based on almost the same spinel "building block", there is an important structural difference between them provided by the occupancy of the octahedral positions (populated with Fe^{3+} ions) and the ordering degree of the cationic vacancies on these positions (1/6 of the octahedral positions). As described by C. Pecharromán et al. in Phys. Chem. Miner. 22, 21–29, 1995, *three possible maghemite varieties* have been evidenced: in the case of a totally random distribution of the Fe vacancies over the octahedral positions, a maghemite (Fe_2O_3) structure having an Fd-3m symmetry (S.G. no. 227) is obtained, just as for a perfect (free of vacancies) spinel structure such as magnetite (Fe_3O_4). If the Fe vacancies are ordered in some particular octahedral positions, two different symmetries can be generated, namely a cubic primitive (S.G. no. 213, P4132) lattice, keeping the same lattice parameter (a = 0.833 nm) or a tetragonal one (S.G. no. 96, P43212). The tetragonal unit cell has the c lattice parameter three times as that of spinel (a = b= 0.8330 nm, c = 2.4990 nm), and ordered Fe vacancies placed in the 5/8, 3/8, 2/24 octahedral positions (8b Wyckoff position).

In our case, some of the analyzed FFT patterns could only be explained by using the maghemite structure while others could be indexed either as magnetite or maghemite. We have mentioned this in the manuscript at the end of section "3. Results and discussion": "*In some other cases, the grain orientation (far from a zone axis) did not allow us to come to a clear conclusion as to their crystal structure, since the observed lattice fringes and the associated FFT patterns could be indexed either as magnetite or maghemite.*"

The experimental data that we obtained by HRTEM, contain undeniable facts like the presence of diffraction spots / FFT maxima in forbidden positions that cannot be explained by the magnetite structure. The grain analyzed in the manuscript was certainly not the only situation of the kind, although it was indeed one of the best orientations that we could obtain among the analyzed grains. Given the structure similarity between magnetite and maghemite, only a limited number of crystal orientations enable the observation of those details (usually additional faint spots) helping to differentiate the two structures.

Beside the example presented in the manuscript, in the series of images hereafter we present other situations which cannot be explained using the magnetite structure. Although the observation conditions are not ideal, due to the amorphous biological environment surrounding the nanocrystals, the FFT diagrams of the HRTEM micrographs contain undeniable evidence that helped us taking a decision regarding the crystal structure of the analyzed grains. The FFT diagram from a large area of the grain in the HRTEM micrograph (Figure S2a) contains strong spots which can be assigned to families of planes in magnetite.

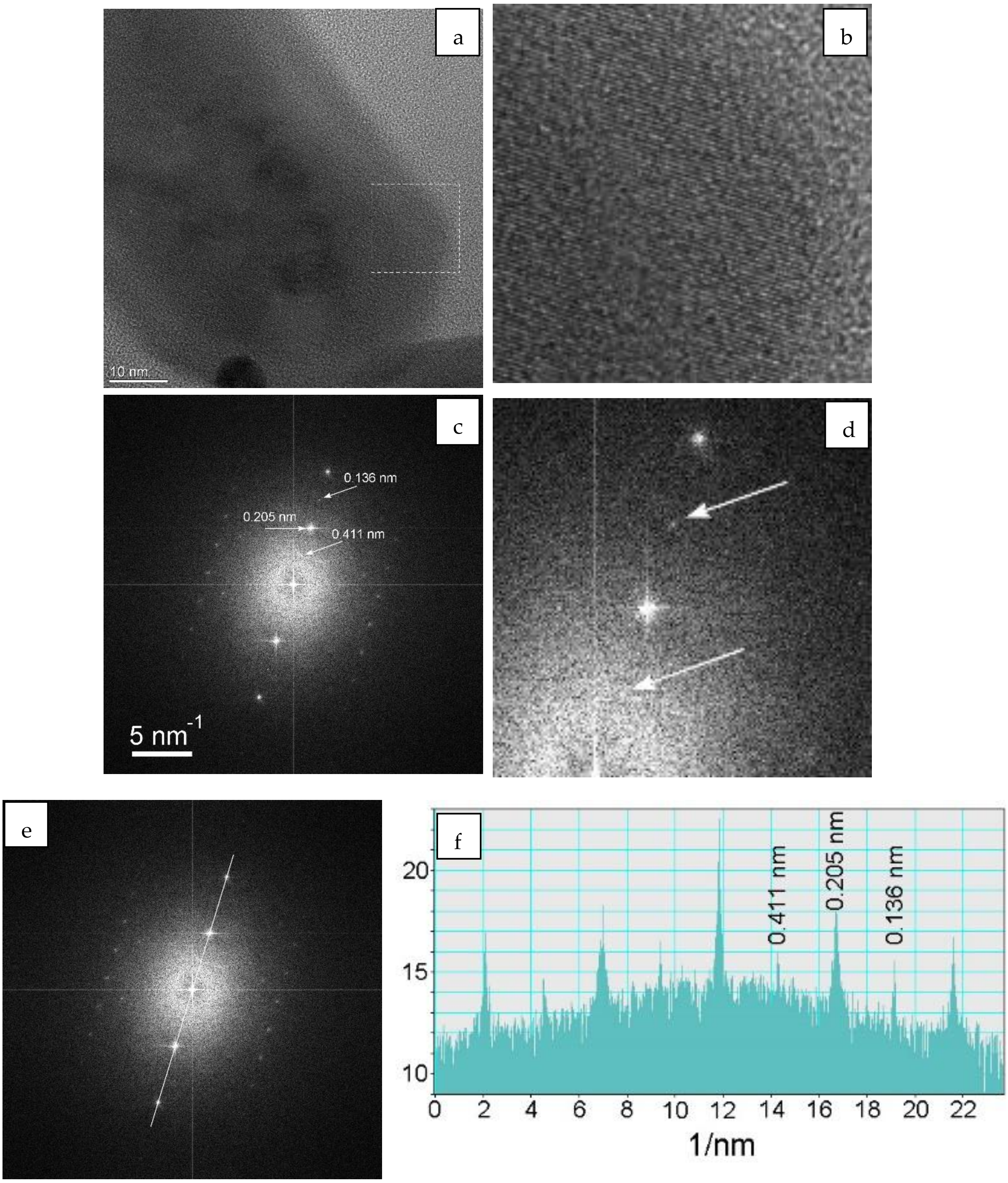

Figure S2. (**a**) HRTEM micrograph of a magnetosome and (**b**) the enlarged image corresponding to the dashed-line square in (**a**) showing the HR lattice planes; (**c**) FFT diagram from a large area on the grain in (**a**) containing faint spots (pointed by tilted arrows) in positions that are not allowed for the magnetite structure; (**d**) enlargement of the FFT in (**c**) showing the faint spots in forbidden positions; (**e**) Orientation of the line profile through the FFT maxima in (**c**) and (**f**) intensity of the FFT maxima along the line profile.

The strong spot indicated by the horizontal arrow corresponds to an interplanar distance of 0.205 nm which can be assigned to the {400} family of planes in magnetite. However, at a careful inspection, the FFT diagram contains also faint spots in half-way positions. For a better visualization of the faint spots, we represented in Figure S2f the intensity profile along a line crossing the FFT maxima (Figure S2e). The faint spots correspond

to interplanar distances of 0.411 nm and 0.136 nm. These spots are not allowed for the ideal magnetite structure, suggesting stoichiometry deviations with an impact on the structure factor. The presence of vacancies would be a cause for the imbalance of the magnetite structure factor, pointing to the related structure of maghemite. And, indeed, these maxima are permitted for the maghemite structure being assigned to {200} and {600} planes, while the strong spot at 0.411 nm would correspond to the (400) planes in maghemite (cif 9012692).

Another example is presented in Figure S3. The FFT pattern of the HR image contain spots that cannot be assigned to allowed reflections for magnetite. The pattern could be reliably indexed using the structure of tetragonal maghemite (cif 9006318).

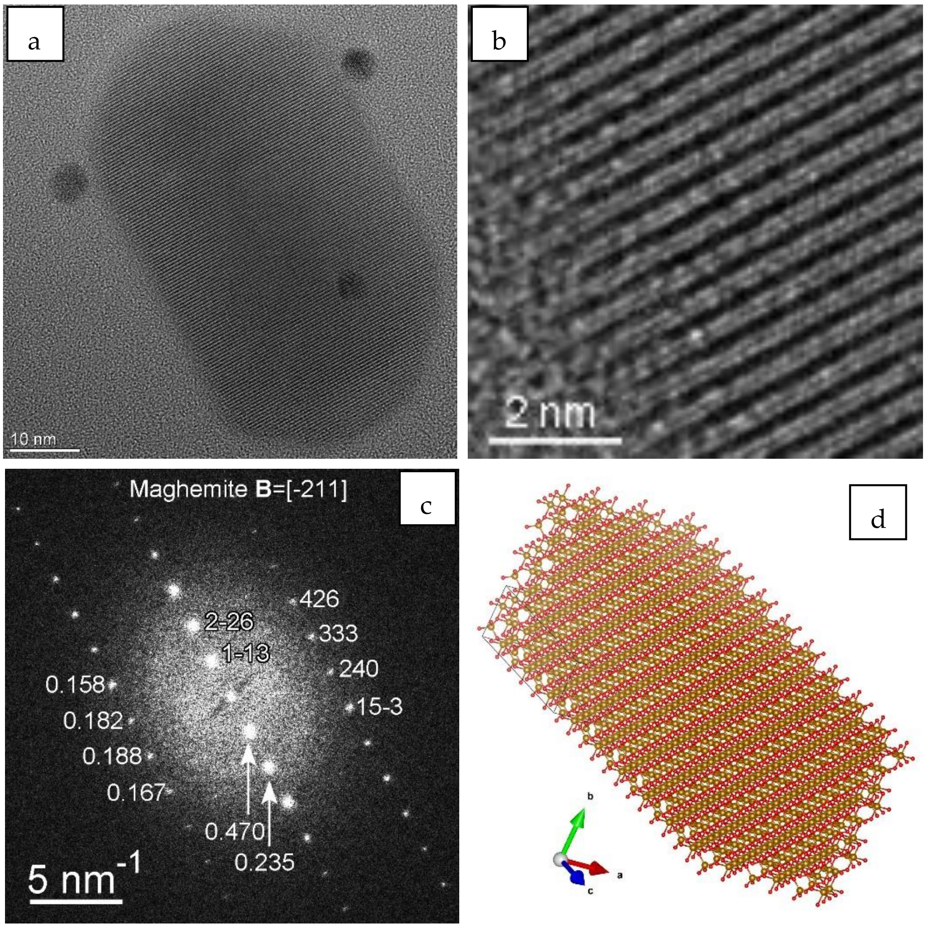

Figure S3. (**a**) HRTEM micrograph of a magnetosome and (**b**) the enlarged image from the left-hand border of the grain (**a**) showing the HR lattice planes; (**c**) FFT diagram from a large area on the grain; the calculated interplanar distances in nm are indicated on the left side of the diagram and the Miller indices according to the maghemite structure on the right side of the diagram; the crystal orientation corresponds to **B** = [−211]; (**d**) the atomic structural model of the tetragonal maghemite in [−211] orientation (obtained with VESTA).

A third example is presented in Figure S4. The FFT pattern associated to the HRTEM image contains details that are incompatible with the magnetite structure. Apart from the strong spots that can be indexed using either of the two structures, fine details may be observed at a careful inspection. The intensity line profile along the dashed line crossing the hh0 spots reveals intensity maxima corresponding to an interplanar distance of 0.58 nm. While extinct for magnetite, this spot is allowed for maghemite (cif 9012692), corresponding to the {110} family of planes. The whole FFT pattern has been indexed accordingly.

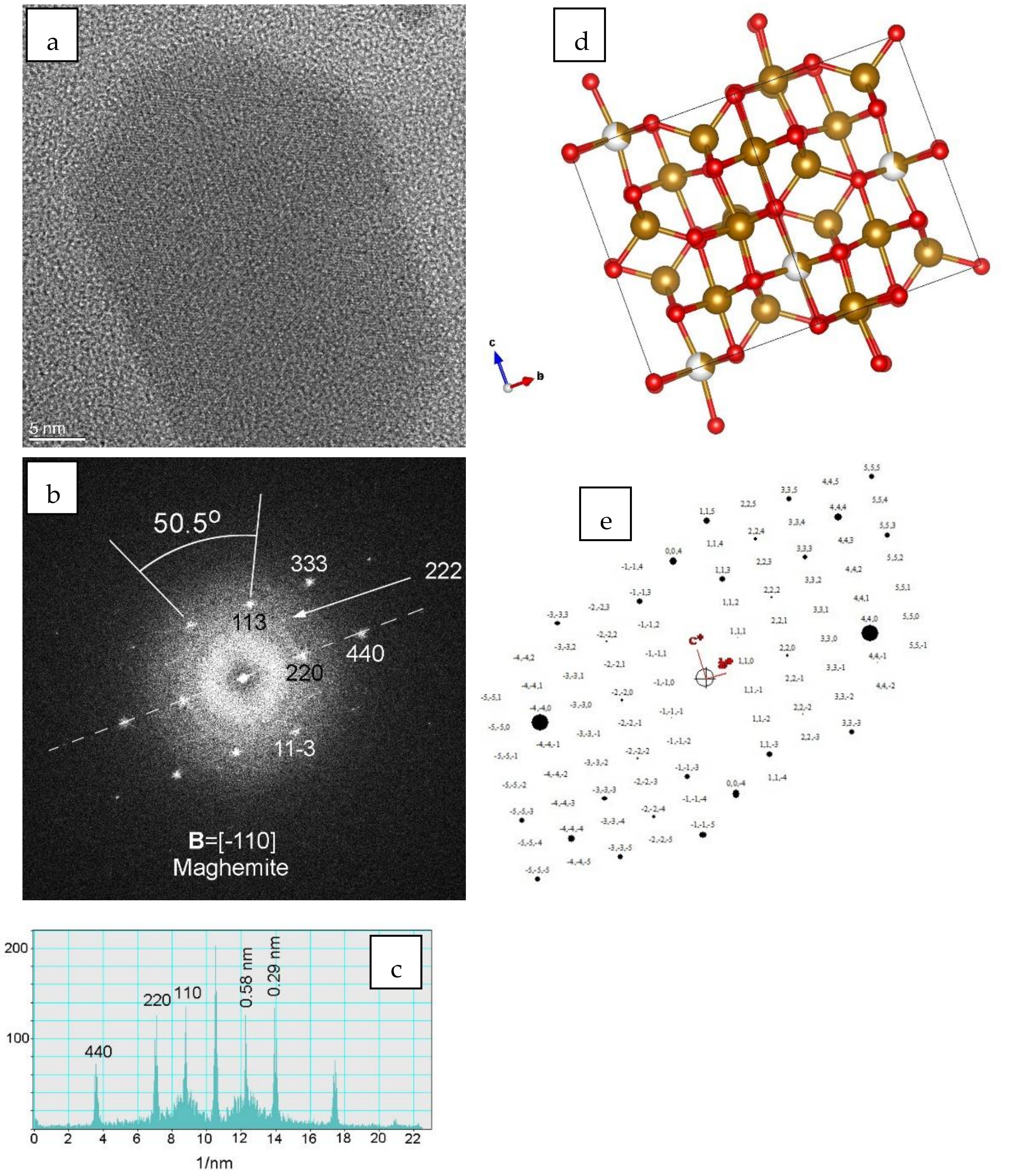

Figure S4. (**a**) HRTEM micrograph of a magnetosome and (**b**) the associated FFT diagram indexed according to the maghemite structure (**B** = [−110]); (**c**) intensity profile along a line crossing the hh0 spots; (**d**) the atomic structural model of maghemite in [−110] orientation (using VESTA); (**e**) simulated electron diffraction pattern using the maghemite structure in [−110] orientation.
